# Drug resistance patterns of *Mycobacterium tuberculosis* complex and associated factors among retreatment cases around Jimma, Southwest Ethiopia

**DOI:** 10.1186/s12889-015-1955-3

**Published:** 2015-07-02

**Authors:** Kedir Abdella, Ketema Abdissa, Wakjira Kebede, Gemeda Abebe

**Affiliations:** Department of Medical Laboratory Science and Pathology, College of Health sciences, Jimma University, Jimma, Ethiopia; Mycobacteriology Research Centre, Institute of Biotechnology Research, Jimma University, Jimma, Ethiopia

**Keywords:** Tuberculosis, MDR-TB, Drug resistance, Drug susceptibility test

## Abstract

**Background:**

Information on the pattern of drug resistant tuberculosis (TB) among re-treatment cases is crucial to develop appropriate control strategies. Therefore, we conducted this study to assess the drug resistance pattern of *M. tuberculosis* complex (MTBC) isolates and associated factors among re-treatment cases in Jimma area, Southwest Ethiopia.

**Methods:**

Health facility-based cross-sectional study was conducted between March 2012 and April 2013 in Jimma area, Southwest Ethiopia. We included 79 re-treatment cases selected conveniently. Socio demographic and clinical data were collected using structured questionnaire. Sputum sample processing, mycobacterial culture, isolation and drug susceptibility testing (DST) were done at Mycobacteriology Research Centre (MRC) of Jimma University. All data were registered and entered in to SPSS version 20. Crude odds ratio (COR) and adjusted odds ratios (AOR) were calculated. P-values less than 0.05 were considered statistically significant.

**Results:**

Seventy-nine re-treatment cases included in the study; 48 (60.8 %) were males. Forty- seven (59.5 %) study participants were from rural area with the mean age of 31.67 ± 10.02 SD. DST results were available for 70 MTBC isolates. Majority (58.6 % (41/70)) isolates were resistant to at least one of the four first line drugs. The prevalence of multidrug-resistant TB (MDR-TB) was 31.4 % (22/70). Place of residence (AOR = 3.44 (95 % CI: 1.12, 10.60), duration of illness (AOR = 3.00 (95 % CI: 1.17, 10.69) and frequency of prior TB therapy (AOR = 2.99, (95 % CI: 1.01, 8.86) were significant factors for any drug resistance. Moreover, history of treatment failure was found to be associated with MDR-TB (AOR = 3.43 (95 % CI: 1.14, 10.28).

**Conclusion:**

The overall prevalence of MDR-TB among re-treatment cases around Jimma was high. The rate of MDR-TB was higher in patients with the history of anti-TB treatment failure. Timely identification and referral of patients with the history of treatment failure for culture and DST need to be strengthened.

## Background

The MTBC is a group of closely related Gram-positive bacilli. The group comprises of the typical human pathogens *M. tuberculosis* and *M. africanum* and variants of pathogens to different animal species [1]. TB in human, caused mostly by *M. tuberculosis*, is recognized as one of the most important threats to human health causing mortality, morbidity and economic losses throughout the world [2].

World health organization (WHO) global report of 2014 ranked Ethiopia as one of the 22 high TB burden countries in the world, with an estimated incidence and prevalence of 258/100,000 and 237/100,000 population, respectively [3]. Moreover, the Ethiopia Ministry of Health hospital statistics data show that TB is the third leading cause of outpatient morbidity and mortality and the fourth leading cause of hospital admission [4].

The burden of TB accompanied with the emergence of drug resistance in clinical settings is a well-recognized problem. MDR-TB, defined as TB caused by strains of MTBC that are resistant to at least rifampicin and isoniazid, is public health problem. Patients can be infected with drug resistant TB from index patients of a primary drug resistance or drug susceptible MTBC strains can develop resistance to anti-TB drugs resulting in acquired drug resistance. Acquired drug resistance is observed often among re-treatment cases since these groups are more likely to harbor strains with full or partial drug resistance for drugs used in previous treatment [5, 6].

In Sub-Saharan Africa the  rate of MDR-TB is five times higher among previously treated TB cases than new cases [7]. Studies have demonstrated that nearly half million cases of MDR-TB emerged every year [8]. However, only 3 % of these are treated and 110,000 die annually. Moreover, approximately 5-10 % of MDR-TB cases are extensively drug resistant (MDR-TB strains resistant to any of the fluoroquinolones and at least one of the three injectable second line drugs (Amikacin, Kanamycin and/or Capreomycin) [9].

Ethiopia is one of the 27 high MDR-TB burden countries from the world with an estimated prevalence of 1.6 % and 12 % among new and re-treatment cases, respectively [3]. There are a number of factors associated with MDR-TB disease development particularly in rural areas of the country. As a result, the proportion and risk of MDR-TB infection is estimated to be high. Despite the rising public health concern of MDR-TB in Ethiopia, the prevalence and associated factors for the spread of this disease are not well documented. Therefore, the aim of this study was to determine the prevalence and risk factors for drug resistant TB in Jimma area, Southwest Ethiopia.

## Methods

### Study design, area and period

Health facility based cross-sectional study was conducted from March 2012 to April 2013 at MRC of Jimma University. The MRC provides MTB culture and DST to inpatient and outpatients from Jimma University Specialized Hospital and referrals from other health facilities in Southwest part of Ethiopia. All presumptive MDR-TB cases referred from all health facilities in Jimma area to the MRC were included in the study. All the laboratory works were done at Mycobacteriology laboratory of the MRC.

### Study population

The study population were all consecutive smears positive pulmonary tuberculosis re-treatment cases (who had the history of previous anti-TB treatment for at least one month) visiting Jimma University MRC during the study period.

### Exclusion criteria

Patients with the following characteristics were excluded from the study: patients, who were on anti-TB treatment for less than one month at the time of data collection, patients who were less than 15 years old and patients who provided inadequate specimen for the laboratory analysis.

### Sample size

The sample size was calculated based on the sampling method recommended by WHO for drug resistance surveys in TB [10]. The assumptions considered were: total number of previously treated smear positive TB cases registered during 2011 at Jimma zone and Jimma town were 74, the proportion of rifampicin resistance among re-treatment cases was 15.6 % (unpublished data from the MRC of Jimma University), the desired precision of 2 % at 95 % confidence level, z value of 1.96 and a non-response rate of 20 %. Therefore, the final sample size calculated was 84.

### Data collection

Structured questionnaires were used to obtain data on patients’ socio-demographic characteristics and risk for TB drug resistance. HIV sero-status prior to enrollment was collected from patient’s clinical record.

### Laboratory procedures

#### Sputum collection & transportation

Each eligible patient who signed written consent provided sputum specimen independent of the routine samples used for diagnostic purposes to minimize chances of contamination. For each case, sputum smear was prepared and examination was conducted at Mycobacteriology Laboratory of MRC.

#### Sputum culture and drug susceptibility testing (DST)

At the MRC Mycobacteriology Laboratory, all the collected specimens were decontaminated using 1.5 % NaOH-NALC method. All processed samples cultured on liquid media using the BACTEC MGIT 960 TB detection system (Becton Dickinson, USA). A culture was reported negative only if there was no growth after 42 days of incubation. Identification of *M. tuberculosis* was based on para-nirobenzoic acid inhibition test [11]. Phenotypic DST of the isolates against rifampicin, isoniazid, ethambutol and streptomycin was done using the BACTEC MGIT 960 indirect proportion method. The drugs were used at concentrations of 1.0 μg/ml for rifampicin, 0.1 μg/ml for isoniazid, 5.0 μg/ml for ethambutol and 1.0 μg/ml for streptomycin [12].

### Data analysis and interpretation

Data were analyzed using SSPS version 20. Patients’ information were cleaned and double entered into the statistical package. The distribution of drug resistance patterns of MTBC were expressed using tables and charts. The differences in the proportion of drug resistance between groups were compared using chi-square test. Evaluation of risk factors for drug resistance in MTBC among retreatment cases were undertaken by binary logistic regression. Finally multivariate logistic regression analysis were undertaken by including factors found to be significant or marginally at p < 0.25 in binary logistic analysis. P < 0.05 was considered as statistically significant.

### Ethics

Ethical approval was obtained from the ethical review board at the Jimma University College of Health Sciences. Written consent was obtained from every study participant. The results from laboratory analysis were communicated to the responsible physician for early initiation of anti-TB treatment.

## Results

### Baseline characteristics

From 79 re-treatment cases enrolled in the study, 60.8 % (48/79) were males. Most (84.4 % (67/79) were in age group 15-44 years. The age range of the study participants was 15–65 years with mean age of 31.67 + 10.02 years. The majority participants (59.5 % (47/97)) lived in Jimma town and 40.5 % (32/79) of the participants were referred to the Jimma University MRC for TB culture and DST out of Jimma town. Three retreatment sub-categories of cases were involved in this study: 46.8 % (37/79) relapse, 43 % (34/79) treatment failure and 10.1 % (8/79) defaulters. Of the total 79 patients, 97.5 % (77/79) had sputum smear examination and 59.5 % (47/79) had chest X-ray examination before this episode. Thirty-six patients (45.6 %) had experienced ≥ 2 episodes of treatment for TB prior to the current episode and 15.2 % (12/79) cases had TB related injection for more than one month. HIV testing was positive in 29.1 % (23/79) of the study participants and 69.6 % (16/23) of these were on anti-retroviral therapy.

### MTB culture, identification and patterns of drug resistance

**Figure 1:**
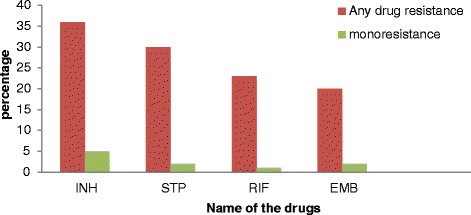
Drug resistance patterns of *M. tuberculosis* complex isolates to each of the five first-line anti- tuberculosis among study participants around Jimma, Southwest Ethiopia from March 2012 to April 2013. INH - Isoniazid, STP - Streptomycin, RIF - rifampicin, EMB- ethambutol

Out of the 79 sputum specimens processed for culture, 89.9 % (71/79) were culture positive. Most of the isolates (98.6 % (70/71)) were identified as MTBC and DST results were available for all MTBC isolates. Isolates from 58.6 % (41/70) patients were resistant to at least one of the four first line drugs. The rate of any resistance to isoniazid and rifampicin was 51.4 % (36/70) and 32.9 % (23/70), respectively (Fig. 1). Out of the 23 rifampicin resistant isolates, 22 were also resistant to isoniazid which resulted in 31.4 % (22/70) MDR-TB prevalence. Twelve (17.1 %) of the 22 MDR-TB isolates were resistant to all first-line drugs. Overall, 14.3 % (10/70) were mono drug resistant (resistant to only one of the four tested drugs) and 12.9 % (9/70) were poly drug resistant (resistant to two or more drugs without the combination of isoniazid and rifampicin) (Table 1).

**Table 1:** Pattern of drug resistance in *M. tuberculosis* complex isolates from retreatment cases around Jimma, Southwest Ethiopia from March 2012 to April 2013 (n = 70)

Drug resistance patterns	Frequency n (%)
Any drug resistance	41 (58.6)
INH^a^	36 (51.4)
RIF^b^	23 (32.9)
STP^c^	30 (42.9)
EMB^d^	20 (28.6)
Two drugs resistance	
INH + STP	4 (5.7)
INH + EMB	2 (2.9)
STP + EMB	1 (1.4)
Three or more drugs resistance	
INH + RIF + STP	9 (12.9)
INH + RIF + EMB	1 (1.4)
INH+ STP + EMB	2 (2.9)
INH + RIF + STP+ EMB	12 (17.1)
MDR-TB^e^	22 (31.4)
Poly drug resistance^f^	9 (12.9)
Mono drug resistance^g^	10 (14.3)

INH^a^ = Isoniazid, RIF^b^ = Rifampicin, STP^c^, = Streptomycin, EMB^d^ = Ethambutol, MDR^e^ = Multi drug resistance (resistance at least INH and RIF), Poly drug^f^ = resistance to two and more drugs without the combination of INH and RIF, monodrug^g^ = single drug resistance

### Factors associated with drug resistance

In a multivariable analysis, it was found that place of residence, (AOR = 3.44 (95 % CI: 1.12, 10.60), duration of illness (AOR = 3.00 (95 % CI: 1.17, 10.69) and frequency of prior TB therapy (AOR = 2.99, (95 % CI: 1.01, 8.86) were significant factors to any drug resistance (Table 2). Patients with history of treatment failure or defaulters were more likely to have isolates with rifampicin resistance (AOR = 3.04, (95 % CI: 1.02, 9.10) or MDR-TB (AOR = 3.43 (95 % CI: 1.14, 10.28). All the rifampicin resistant isolates were MDR-TB. Risk factors appeared to differ by subgroup, though analyses were limited by sample size (Table 3 and Table 4).

**Table 2:** Univariate and multivariate analysis of risk factors for any drug resistance in *M. tuberculosis* complex isolates from retreatment cases around Jimma, Southwest Ethiopia from March 2012 to April 2013 (n = 70)

Variables	Any drug resistance		COR (95 % CI)	p-value	AOR (95 % CI)	p-value
	Yes	No				
Residence place						
Urban	22 (68.8 %)	10 (31.2 %)	2.3 (1.83, 5.87)	0.015	3.44 (1.12, 10.60)	0.032
Rural	19 (50 %)	19 (50 %)	1		1	
Number of treatment before this episode						
Two or more time	2 (68.8 %)	10 (31.2 %)	2.2 (1.72, 5.89)	0.021	2.99 (1.01, 8.86)	0.048
One time	19 (50 %)	19 (50 %)	1		1	
Duration of illness for this episode						
≥3 months	30 (65.2 %)	16 (34.8 %)	2.26 (1.81, 6.10)	0.016	3.00 (1.17, 10.69)	0.039
≤2 months	11 (45.8 %)	13 (54.2 %)	1		1	

*COR* Crude Odds Ratio, *AOR* Adjusted Odds Ratio

**Table 3:** Univariate and multivariate analysis of risk factors for RIF resistance in *M. tuberculosis* complex isolates from retreatment cases around Jimma, Southwest Ethiopia from March 2012 to April 2013 (n = 70)

Variables	Rifampicin resistance		COR (95 % CI)	p-value	AOR (95 % CI)	p-value
	Yes	No				
HIV sero-status						
Positive	11 (47.8 %)	12 (52.2 %)	2.67 (0.937, 7.63)	0.066	2.89 (0.97, 8.64)	0.057
Negative	11 (23.4 %)	36 (76.6 %)	1		1	
Retreatment sub-categories						
Treatment failure and defaulters	16 (43.2 %)	21 (56.8 %)	2.8 (0.98, 8.15)	0.054	3.04 (1.02, 9.10)	0.047
Relapse	7 (21.2 %)	26 (78.8 %)	1		1	
Alcohol abuse						
Yes	5 (55.6 %)	4 (44.4 %)	2.99 (0.72, 12.42)	0.143	2.89 (0.61, 13.58)	0.180
No	18 (29.5 %)	43 (70.5 %)	1			
History of being in prison						
Yes	6 (50 %)	6 (50 %)	2.41 (0.68, 5.55)	0.189**	1.8 (0.43, 7.57)	0.421
No	23 (39.7 %)	35 (60.3 %)	1		1	

*COR* Crude Odds Ratio, *AOR* Adjusted Odds Ratio

**Fisher’s Exact Test p-value

**Table 4:** Univariate and multivariate analysis of risk factors for MDR-TB in *M. tuberculosis* complex isolates from retreatment cases around Jimma, Southwest Ethiopia from March 2012 to April 2013 (n = 70)

Variables	MDR-TB		COR (95 % CI)	p-value	AOR (95 % CI)	p-value
	Yes	No				
HIV sero-status						
Positive	10 (43.5 %)	13 (56.5 %)	2.24 (0.78, 6.43)	0.133	2.3 (0.74, 7.14)	0.151
Negative	11 (23.4 %)	36 (76.6 %)	1		1	
ART status						
Retreatment sub-categories						
Treatment failure and defaulter	16 (43.2 %)	21 (56.8 %)	3.43 (1.14, 10.28)	0.028	3.4 (1.14, 10.28)	0.028
Relapse	6 (18.2 %)	27 (81.8 %)	1		1	
Alcohol abuse						
Yes	5 (55.6 %)	4 (44.4 %)	3.24 (0.78, 13.51)	0.128**	3.4 (0.78, 15.63)	0.106
No	17 (27.9 %)	44 (72.1 %)	1		1	
History of being in prison						
Yes	6 (50 %)	6 (50 %)	2.63 (0.74, 9.34)	0.174**	1.2 (0.46, 8.36)	0.359
No	16 (27.6 %)	42 (72.4 %)			1	

*COR* Crude odds ratio, *AOR* Adjusted odds ratio

**Fisher’s Exact Test p-value

## Discussion

In this study, the drug resistance patterns of MTBC and associated factors were evaluated. The overall resistance to one or more first line drug(s) was 58.6 %. This was in agreement with the previous study in Addis Ababa in which 58 % of isolates were resistant to one or more drugs [13]. However, any drug resistance rate in the present study was lower than the rate observed in recent study in Addis Ababa where 72.9 % isolates were resistant to one or more drugs [14]. This difference in findings might be due to difference in proportion of retreatment sub-categories since majority of the cases in that study were referral cases and most referral cases were treatment failure.

The current WHO treatment guideline recommends the combined treatment of two months rifampicin, isoniazid, pyrazinamide, ethambutol and streptomycin, one-month rifampicin, isonaizid, pyrazinamide, ethambutol and, five months rifampicin, isonaizid, and ethambutol (2RHZES/RHZE/5RHE) for previously treated TB patients by simply adding streptomycin to the regimen for new cases. This repeated treatment practice can amplify resistance in these patients who are likely to have developed resistance to some or all of the previously used first line anti- TB drugs [15]. The situation is further complicated in developing countries where there are inadequate laboratories that have capacity for culture and DST. This challenge could be surmounted by giving priority for previously treated TB cases for culture and DST.

The highest proportion of any drug resistance was observed to isoniazid (51.4 %). This is comparable with the study done in India (52 %) [16] and recent report from study in Addis Ababa (56.1 %) [14]. However, our finding was higher than that of previous studies in Ethiopia 44 % [13] and 42.7 %) [17]. The higher prevalence of isoniazid resistance has also important implications. Isoniazid is the cornerstone drug used throughout the course of non-MDR-TB treatment. It is also the drug of choice for chemoprophylaxis of TB in developing countries for treating latent TB infection. Loss of the effectiveness of this drug compromises both the preventive therapy and treatment of TB disease. Moreover, it is predictor for MDR-TB in the future since MDR-TB often develops from initial isoniazid mono-resistant strains [18].

The second highest any resistance was against streptomycin (42.9 %). The figure is high when compared with earlier studies in Ethiopia where streptomycin resistance accounted for 21 % [17] and 28 % [13]. However, the result is lower than that of recent study in Addis Ababa (67.3 %) [14]. The high any resistance to streptomycin may be due to its early introduction, its common use for treatment of other bacterial infections and inadequate treatment due to poor compliance by patients [19].

The rate of rifampicin resistance was 32.9 %. This is in agreement with the study in Addis Ababa (33.3 %) [13]. The higher rate of rifampicin resistance might be due to its adverse effects such as nausea, vomiting, rashes, hepatitis, GIT upset, flu-like symptoms, fever and jaundice, which could result in patient non-adherence and hence may lead to the selection of resistant strains [19]. In this study, there was only one case with rifampicin mono resistance. The low proportion (1.4 %) of non-MDR rifampicin resistance in this study supports the use of rifampicin resistance as surrogate marker for MDR-TB. It is also in line with WHO recommendation of non MDR-TB rifampicin resistance less than 3 % as good quality performance indicator [20].

The proportion of any ethambutol resistance was 28.6 %. Ethambutol is the first-line drug included in the regimen of second-line drugs to treat MDR-TB cases. The high rate of ethambutol resistance would challenge its inclusion in MDR-TB therapy as this may lead to unintentional incorrect therapy [21]. Thus, further study is recommended to know the level of ethambutol resistance specifically in MDR-TB isolates. This can help in developing national or regional standardized second-line treatment regimen for MDR-TB cases.

In this study the prevalence of MDR-TB (31.4 %) was more than twice the Ethiopian national prevalence of 12 % for retreatment cases [3]. The rate of MDR-TB in retreatment cases in this study is higher than other studies conducted in Uganda (17.8 %) [22] and Ethiopia (28 %) [13]. However, our finding is lower than the recent report from Addis Ababa (46.3 %) [14], India (47.1 %) [16] and Philippines (76.4 %) [23]. The discrepancy in findings between the present study and that of recent study in Addis Ababa can be explained by the differences in the nature of the populations included in the studies. The study in Addis Ababa was conducted retrospectively on data from national TB diagnosis and treatment centers. Most of the cases were referral cases. Most of the referral cases for DST and culture in Ethiopia are treatment failures. However, our study included both referral patients and smear positive previously treated cases diagnosed at Jimma University MRC. In addition there may also be geographical variation in the level of drug resistance.

In the present study, majority (90.9 %) of the MDR-TB cases were in the age between 15 and 44 years. This is in agreement with the report from Iran [24]. The high frequency of MDR-TB among young age groups may indicate the likelihood of propagation of MDR-TB in the community because of high mobility of youth from place to place. This also suggests the occurrence of recent transmission of TB infection because the rate of TB in the older age group mostly suggests the infection has been acquired in the past [25].

The origin drug resistant TB is mostly due to chromosomal alterations such as mutations or deletions. However, TB service related factors have a significant impact on the amplification and transmission [26]. Other mechanisms like efflux pumps have their own contribution in drug resistant TB [11]. In the current study, place of residence, duration of illness and frequency of treatments before this episode showed significant association with any drug resistance. In this study, the history of category I and category II treatment failures were identified as the strongest predictors for either rifampicin resistance or MDR-TB. This is consistent with previous studies in Addis Ababa, Ethiopia [13, 14] and China [25]. Our result showed that more than half of treatment failures were identified as MDR-TB. Majority of these cases were from category I treatment failures (75.6 %). This suggests the importance of early request for culture and DST than awaiting the outcome of extended category II treatment among patients for whom category I failed.

High rates of MDR-TB among treatment failures (72.7 %) can be influenced by the acquisition of resistance in the intensive and continuation phases of treatment or the rate of primary MDR-TB infection [27]. However, the rate of MDR-TB among newly diagnosed TB patients in Jimma was low (1.5 %) [28]. Therefore, the most possible reason for higher rate of MDR-TB in our study is acquisition of drug resistance during the intensive or/and continuation phases of treatment. This may provide clue for the importance of evaluation of currently available TB control programs on proper usage of the drugs. Moreover, it supports the necessity of looking in to the adherence of patients to full course of chemotherapy.

Our study suggests that patients from urban area were more likely to harbor drug resistant TB bacilli. The slumps in urban areas are the favorable environment for the TB transmission including the drug resistant strains. Moreover, relatively high access to unregulated antibiotics in urban area may contribute to the development and selection of drug resistant MTB strains. There was high frequency of MDR-TB cases among patients with the history of alcohol drinking and HIV infection. We did not find statistically significant association between MDR-TB and alcohol consumption and HIV positivity. However, alcohol consumption and HIV treatment can cause concomitant hepatotoxicity that may lead to inadequate adherence to the anti-TB treatment.

This study has its own limitations. Frist, it is institution-based study and hence there could have been significant referral bias involved in patient selection. Second, since DST was not recorded in the previous disease episode, we were unable to determine the extent of amplification in the acquired drug resistance in study population. Finally, data about people’s contact with MDR-TB cases were not available. Despite this limitation, this study provided the first information on TB drug resistance among previously treated cases in the study setting. This can be used for better planning of TB management and tackling further increase in the level of MDR-TB.

## Conclusion

High prevalence of any drug resistance and MDR-TB were detected among previously treated cases around Jimma. The proportion of MDR-TB was significantly higher among patients with the history of treatment failures. TB patients with history of treatment failures should timely be identified and referred for culture and DST.

## References

[1] Karlson GA, Lessel EF (1970). Mycobacterium bovis. Nom Nov Int J Sys Bacteriol.

[2] World Health Organization. Global tuberculosis Control report 2010. Available at http://reliefweb.int/sites/reliefweb.int/files/resources/F530290AD0279399C12577D8003E9D65-Full_Report.pdf. Accessed on March 25, 2012

[3] World Health Organization (2014). Global tuberculosis report.

[4] Federal Democratic Ripublic of Ethiopia Minstry of Health (2011). First Ethiopian National Population Based Tuberculosis Prevalence Survey.

[5] GBC-Health. Drug-resistant-TB:-why-it-matters. availble at: http://www.gbchealth.org/system/documents/category_1/7/GBCHealth%20Issue%20Brief_Drug-Resistant%20TB.pdf. Accessed on March 25, 2012.

[6] Mak A, Thomas A, DelGranado M, Zaleskis R, Mouzafarova N, Menzies D (2008). Influence of Multidrug Resistance on Tuberculosis Treatment Outcomes with Standardized Regimens. Am J Respir Crit Care Med.

[7] Berhan A, Berhan Y, Yizengaw D: A meta-analysis of drug resistanttuberculosis in Sub-Saharan Africa: how strongly associated with previous treatment and HIV co-infection? Ethiop JHealth Sci, 23(3):271-282. 10.4314/ejhs.v23i3.10 PMC3847537 24307827

[8] Anthony SF (2008). Multidrug-Resistant and Extensively Drug-Resistant Tuberculosis: The National Institute of Allergy and Infectious Diseases Research Agenda and Recommendationsfor Priority Research. J of Inf Dis.

[9] Falzon D, Jaramillo E, Schünemann HJ, Arentz M, Bauer M, Bayona J (2011). WHO guidelines for the programmatic management of drug-resistant tuberculosis: 2011 update. Eur Respir J.

[10] World health organization. Guidelines for surveillance of drug resistance in tuberculosis-4th ed. WHO/HTM/TB/2009.422. Geneva, Switzerland: World Health Organization; 2009.

[11] Zhang Y, Yew WW (2009). Mechanisms of drug resistance in Mycobacterium tuberculosis. Int J Tuberc Lung Dis.

[12] Canneti G, Fox W, Khomenko A (1969). Advances in techniques of testing mycobacterial drug sensitivity, and the use of sensitivity tests in tuberculosis programmes. Bull World Health Organ.

[13] Meskel DW, Abate G, Lakew M, Goshu S, Aseffa A (2008). Anti-tuberculosis drug resistance among retreatment patients seen at St Peter Tuberculosis Specialized Hospital. Ethiop Med J.

[14] Abate D, Taye B, Abseno M, Biadgilign S. Epidemiology of anti-tuberculosis drug resistance patterns and trends in tuberculosis referral hospital in Addis Ababa, Ethiopia. BMC Res Notes. 2012;5:462. 10.1186/1756-0500-5-462 PMC3507648 22929063

[15] Jones-López EC, Ayakaka I, Levin J, Reilly N, Mumbowa F, Dryden-Peterson S (2011). Effectiveness of the standard WHO recommended retreatment regimen (category II) for tuberculosis in Kampala, Uganda: a prospective cohort study. Plos Med.

[16] Hanif M, Malik S, Dhingra VK (2009). Acquired drug resistance pattern in tuberculosis cases at the state tuberculosis centre, Delhi. India Int J Tuberc Lung Dis.

[17] Abate G, Miörner H, Ahmed O, Hoffner SE (1998). Drug resistance in Mycobacterium tuberculosis strains isolated from re-treatment cases of pulmonary tuberculosis in Ethiopia: susceptibility to first-line and alternative drugs. Int J Tuberc Lung Dis.

[18] Jenkins HE, Zignol M, Cohen T (2011). Quantifying the burden and trends of isoniazid resistant tuberculosis-1994-2009. PLoS ONE.

[19] Gillespie SH (2002). Evolution of Drug resistance in Mycobacterium tuberculosis: Clinical and molecular perspective. Antimicrob Agents Chemother.

[20] World Health Organization. International Union against Tuberculosis and Lung Disease. Anti-tuberculosis drug resistance in the world: 4th global report. available at: http://www.who.int/tb/publications/2008/drs_report4_26feb08.pdf. Accessed on March 25, 2012. 2008.

[21] Hoek KG, Schaaf HS, van Pittius NC, van Helden PD, Warren RM. Resistance to pyrazinamide and ethambutol compromises MDR/XDR-TB treatment. SAMJ. 2009;99:785-87. 20218473

[22] Temple B, Ayakaka I, Ogwang S, Nabanjja H, Kayes S, Nakubulwa S (2008). Rate and Amplification of Drug Resistance among Previously-Treated Patients with Tuberculosis in Kampala. Uganda Clin Infec Dis.

[23] Gler MT, Macalintal LE, Raymond L, Guilatco R, Quelapio MI, Tupasi TE (2011). Multidrug-resistant tuberculosis among previously treated patients in the Philippines. Int J Tuberc Lung Dis.

[24] Merza MA, Farnia P, Tabarsi P, Khazampour M, Masjedi MR, Velayati AK (2011). Anti-tuberculosis drug resistance and associated risk factors in a tertiary level TB centre in Iran: A retrospective analysis. J Infect Dev Ctries.

[25] Liang L, Wu Q, Gao L, Hao Y, Liu C, Xie Y (2012). Factors contributing to the high prevalence of multidrug-resistant tuberculosis: a study from China. Thorax BMJ.

[26] Migliori GB, Dheda K, Centis R, Mwaba P, Bates M, O’Grady J (2010). Review of multidrug-resistant and extensively drug-resistant TB: global perspectives with a focus on sub-Saharan Africa. Trop Med Int Health.

[27] Sharma SK, Kumar S, Saha PK, George N, Arora SK, Gupta D (2011). Prevalence of multidrug-resistant tuberculosis among Category II pulmonary tuberculosis patients. Indian J Med Res.

[28] Abebe G, Abdissa K, Abdissa A, Apers L, Agonafir M, de-Jong BC (2012). Relatively low primary drug resistant tuberculosis in southwestern Ethiopia. BMC Res Notes.

